# Shensu IV prevents glomerular podocyte injury in nephrotic rats via promoting lncRNA H19/DIRAS3-mediated autophagy

**DOI:** 10.1042/BSR20203362

**Published:** 2021-05-04

**Authors:** Yong Huang, Yaqian Huang, Yehua Zhou, Jie Cheng, Chanjun Wan, Maohong Wang, Chiheng Pi, Guoqing Wu, Weiguo Song

**Affiliations:** 1 Department of Nephrology, Affiliated Hospital of Jiangxi University of Traditional Chinese Medicine, Nanchang 330006, China; 2 College of Science and Technology, Jiangxi University of Traditional Chinese Medicine, Nanchang 330004, China; 3 Graduate School, Jiangxi University of Traditional Chinese Medicine, Nanchang 330004, China; 4 Department of Cardiovascular, Affiliated Hospital of Jiangxi University of Traditional Chinese Medicine, Nanchang 330006, China

**Keywords:** autophagy, DIRAS3, lncRNA H19, podocyte damage, Shensu IV

## Abstract

Shensu IV is a Chinese prescription well-known for its function in treating chronic kidney diseases. However, the potential mechanisms underlying how Shensu IV exerts its effects remain unclear. In the present study, we investigated the effects of Shensu IV on glomerular podocyte injury in nephrotic rats and puromycin-induced injury in cultured podocytes, and assessed the associated molecular mechanisms. Liquid chromatography–mass spectrometry (LC–MS) results showed that the main components of Shensu IV were l-Carnitine, P-lysoPC (LPC) 16:0, Coumaroyl tyramine, Tetramethylpyrazine, LPC 18:1, Choline, (S,S)-Butane-2,3-diol, and Scopoletin. We further found that nephrotic rats displayed pathological alterations in kidney tissues and ultrastructural changes in glomerular podocytes; however, these effects were reversed with Shensu IV treatment. Compared with the control, the numbers of autophagosomes were markedly reduced in the model group, but not in the Shensu IV treatment group. Furthermore, the expression of p62 was significantly higher in the model group than in the controls, whereas the LC3-II/I ratio was significantly lower; however, these changes were not observed when Shensu IV was administered. The protective effects of Shensu IV were further confirmed in podocytes displaying puromycin-induced injury. Compared with control group, the expression of long non-coding RNA (lncRNA) H19, mTOR, p-mTOR, and p62 was significantly increased in the puromycin group, whereas that of distinct subgroup of the RAS family member 3 (DIRAS3) was significantly decreased, as was the LC3-II/I ratio. The opposite results were obtained for both shH19- and Shensu IV-treated cells. Collectively, our data demonstrated that Shensu IV can prevent glomerular podocyte injury in nephrotic rats and puromycin-treated podocytes, likely via promoting lncRNA H19/DIRAS3-regulated autophagy.

## Introduction

Podocytes are specialized epithelial cells occupying the visceral layer of the renal glomerular corpuscle. Together with the basement membrane and glomerular endothelial cells, they constitute the glomerular filtration barrier [1]. Podocytes play an important role in the renewal and repair of the glomerular membrane, the regulation of intrinsic glomerular cell function, and immune responses [2]. They can also regulate other glomerular cells by secreting soluble factors and coordinate signaling transduction in the septum of the hiatus to maintain the normal function of the glomerular filtration barrier [3]. Injury to podocytes or their loss is a leading cause of kidney diseases [4].

Autophagy is a lysosome-dependent catabolic pathway in eukaryotic cells. In the physiological state, a basal level of autophagy is required for the removal of unfolded or misfolded proteins and for protein degradation for amino acid production, among others, so as to maintain the stability of the intracellular environment [5]. Although autophagic cell death plays an important role in disease occurrence and development [5], a moderate amount of autophagy is required for kidney development and podocyte differentiation, and an imbalance of autophagy is closely related to the occurrence of glomerular lesions, IgA nephropathy, idiopathic membranous nephropathy, and other diseases [6,7].

Long non-coding RNAs (lncRNAs) are nonprotein coding RNA molecules longer than 200 nucleotides. Although it was initially thought that lncRNAs had no biological function [8], it is now known that they play important roles in epigenetic, transcriptional, and post-transcriptional regulation, as well as in protein metabolism [9]. Interestingly, lncRNAs are also reported to be involved in the regulation of autophagy [10]. LncRNA H19 is a widely investigated lncRNA that plays an important role in the regulation of stem cell differentiation [11] and cancer cell growth [12], and is also a known driver of uterine leiomyomas [13].

Distinct subgroup of the RAS family member 3 (*DIRAS3*), also known as aplasia Ras homolog member I, is a maternally imprinted tumor suppressor gene that in humans is located on Chromosome 1p31. *DIRAS3*, which encodes a small GTP-binding protein, is widely expressed in epithelial cells of various organs, including the ovary and breast, while it is down-regulated or absent from several cancers, including ovarian cancer, breast cancer, and pancreatic cancer [14]. However, whether DIRAS3 is involved in nephropathy is still not known.

Shensu IV is a Chinese prescription well-known for its function in treating chronic kidney diseases. The main components of Shensu IV include raw *Astragalus gummifer* Labill., stir-fried *Angelica sinensis* (Oliv.) Diels, *Stephania tetrandra* S.Moore, *Sinomenium acutum* (Thunb.) Rehder and E.H.Wilson, medicinal silkworms, *Paris polyphylla* var. *chinensis* (Franch.) H.Hara, and *Lycopus lucidus* var. *hirtus* (Regel) Makino and Nemoto. We have previously reported on the function of Shensu IV against podocyte injury in rats with puromycin-induced nephropathy [15]; however, the mechanisms involved remain largely unknown. To provide a theoretical basis for the prevention and treatment of podocyte injury in chronic kidney disease, we sought to identify the regulatory mechanism underlying the effects of Shensu IV on the injury of podocytes.

## Materials and methods

### Preparation of Shensu IV decoction

All the constituents of Shensu IV were purchased from Jiangzhong Decoction Company (Nanchang, China) and included 30 g of raw *Astragalus gummifer* Labill. (Batch No. 200615); 10 g of stir-fried *Angelica sinensis* (Oliv.) Diels (Batch No. 200618); 10 g of *Stephania tetrandra* S.Moore (Batch No. 200531); 15 g of *Sinomenium acutum* (Thunb.) Rehder and E.H.Wilson (Batch No. 200414); 10 g of medicinal silkworms (Batch No. 200513); 10 g of *Paris polyphylla* var. *chinensis* (Franch.) H.Hara (Batch No. 200712); and 15 g of *Lycopus lucidus* var. *hirtus* (Regel) Makino and Nemoto (Batch No. 200205). The medicine was decocted three times and concentrated into a 100-ml stock solution (1 g of crude drug/ml). The solution was sealed in a sterile bottle and stored at 4°C for use.

### Ultra-high-performance liquid chromatography–mass spectrometry

The derived solution was analyzed using an Agilent 1290 UHPLC system as previously described [16]. A 2-μl sample volume was injected into an ACQUITY UPLC HSS T3 column (1.8 μm; 2.1 × 100 mm). The mobile phase included 0.2% formic acid and acetonitrile. The flow rate was 0.2 ml/min and the column temperature was 45°C.

The mass spectrometry (MS) analysis was carried out using an Agilent 6545 Q-TOF. Electrospray ionization (ESI) was conducted in both positive and negative ion modes under the following conditions: nebulizer gas pressure, 4.0 bar; dry gas flow rate, 8 l/min; dry gas temperature, 320°C; ion accumulation time, 0.15 s; time-of-flight, 0.6 ms; capillary voltage, 4.0 kV (positive ion mode), 3.5 kV (negative ion mode). Full-scan MS data were acquired at the mass range of *m/z* 100–3000 amu. Both MS/MS boost and MS/MS isolation modes were selected in the present study.

### Establishment of a rat model of nephropathy

Sixty-six male Sprague–Dawley (SD) rats (weighing 90–110 g) were purchased from Hunan Slake Jingda Experimental Animal Co., Ltd (License No.: scxk [Hunan] 2016-0002). Sixty rats were used for animal modeling and six for the isolation of primary podocytes. All experiments were carried out in Jiangxi University of Chinese Medicine. The animal experimental protocols were approved by the Ethics Committee of Jiangxi University of Chinese Medicine.

The rat nephropathy model was generated as previously reported [17]. Briefly, the rats were anesthetized with 1% pentobarbital sodium (100 mg/kg) and fixed in a supine position. The skin was locally sterilized with 75% ethanol and a 2-cm incision was made along the direction of the jugular vein. The subcutaneous tissue layer was separated to expose the jugular vein. A puromycin aminonucleoside (PAN) solution (10 mg/100 g body weight) was injected through the jugular vein, after which the incision was sutured in layers.

Sixty male SD rats were randomly divided into the following five groups after 1 week of adaptive feeding (*n*=12 per group): a normal control group; a model group; a model + low dose of Shensu IV group; a model + medium dose of Shensu IV group; and a model + high dose of Shensu IV group. Shensu IV solution at the dose of 0.9 ml/(200 g body weight.day^−1^), 1.8 ml/(200 g body weight.day^−1^), or 3.6 ml/(200 g body weight.day^−1^) was administered by gavage at a fixed time everyday from the first day of modeling and for 3 or 6 consecutive weeks. After 3 or 6 weeks, the rats were anesthetized with isoflurane (5%) and killed by decapitation, followed by the collection of kidney tissues.

### Hematoxylin and Eosin, Masson’s, and Periodic Acid–Schiff staining

Kidneys were fixed in 4% paraformaldehyde (PFA) overnight and dehydrated in 70, 80, and 90% ethanol, and then placed in ethanol and xylene for 15 min, xylene I for 15 min, and xylene II for 15 min (until transparent). The kidneys were then placed in a mixture of xylene and paraffin for 15 min, paraffin I and paraffin II for 50 and 60 min, respectively, embedded in paraffin, and sectioned into 10-μm slices. The paraffin sections were baked, dewaxed, and hydrated, and then placed in an aqueous solution of Hematoxylin for 3 min, transferred to a hydrochloric acid ethanol differentiation solution for 15 s, and finally stained with Eosin for 3 min. The samples were sealed and examined under a light microscope.

The sections were dewaxed to water, stained with the prepared Weigert’s Hematoxylin staining solution for 10 min, then with Masson’s blue for 5 min, and finally counterstained with Ponceau Fuchsin staining solution for 8 min. The slides were sealed and examined under a light microscope.

After dewaxing to water, the slides were washed twice with distilled water (1 min each wash), oxidized in periodate solution at room temperature for 7 min, placed in the dark at room temperature for 15 min, and finally transferred to a Hematoxylin staining solution for 2 min. The slides were sealed and examined under a light microscope.

The relative collagen volume fraction and mesangial index were calculated as previously described [18]. Briefly, the collagen positive area (Blue) in Masson’s staining was calculated using ImageJ software. The relative collagen volume fraction was calculated by the formula: The relative collagen volume fraction = collagen positive area (blue)/total area. Quantitative analysis of Periodic Acid–Schiff (PAS) staining was based on the formula: Mesangial index = PAS positive area/total glomerular area.

### Quantitative real-time reverse transcription-PCR

RNA was extracted using TRIzol reagent (CW0580S, CwBio). cDNA was synthesized using a reverse transcription kit (CW2569M, CwBio) and used as a template for fluorescence qPCR. The *GAPDH* gene was used as an internal reference to calculate the expression of lncRNA H19 and *DIRAS3* in each group as previously described [19]. The primers used for qPCR are listed in Table 1.

**Table 1:** Primer sequences

Genes	Primer sequences (5′–3′)	Primer length (bp)	Product length (bp)	Annealing (°C)
*lncRNA H19 F*	CACTACCTGCCTCAGGAATCG	21	137	60.5
*lncRNA H19 R*	CACTACCTGCCTCAGGAATCG	20		
*DIRAS3 F*	ATCGAAGTAAACCGGGCTCC	20	132	60.0
*DIRAS3 R*	CTCGTTGCAGACGCTGTAGA	20		
*β-actin F*	GCCATGTACGTAGCCATCCA	20	375	59.5
*β-actin R*	GAACCGCTCATTGCCGATAG	20		

### Transmission electron microscopy

Tissues were fixed in 2.5% glutaraldehyde, dehydrated, embedded, solidified, and sectioned into 70-nm slices. The tissues were then stained using 3% uranyl acetate and lead citrate, and imaged by transmission electron microscopy (80 kV, JEOL JEM-1230, Tokyo, Japan).

### Preparation of rat podocytes

Rats were killed by decapitation following anesthesia with 5% isoflurane. The kidneys were immediately removed under aseptic conditions and immersed in PBS. The tissues were digested with 0.1% collagenase IV for 10–15 min. The digestion was terminated by the addition of complete culture medium (DMEM, 10565018, GIBCO), and the cells were collected through a 100-μm screen filter. The filtrates were centrifuged at 1000 rpm for 5 min and the collected cells were resuspended in DMEM supplemented with 10% fetal bovine serum and cultured in an incubator with 5% CO_2_ at 37°C.

### Immunofluorescence

The mounting cells were fixed in 4% PFA for 15 min, and then washed again three times with PBS (3 min each wash). The cells were permeabilized with 0.5% Triton X-100 (in PBS) at room temperature for 20 min. After blocking with 5% BSA at 37°C for 30 min, the cells were incubated with a primary antibody against NPHS1 (1:200) at 37°C for 3 h, followed by incubation with a fluorescent secondary antibody (Cy3; 1:200) at 37°C for 45 min. Finally, the nuclei were counterstained with DAPI in the dark for 5 min, and the samples were sealed in 50% glycerol. Images were obtained using a fluorescence microscope.

### Cell transfection and grouping

Cells were transfected when they reached 70% confluence. Polybrene (H8761, Solarbio) (1 μl) was added to 1 ml of cell culture medium. Cells were transduced with lentivirus at a multiplicity of infection (MOI) of 100 and cultured at 37°C for approx. 16 h. The lentivirus encoding an shRNA targeting lncRNA H19 (shH19) was produced by Shanghai Zhonghong Boyuan Biological Technology Co., Ltd (Shanghai, China). After 16 h, the transfection medium was replaced with fresh culture medium. Gene expression was verified after 48 h. The cells were divided into the following groups: blank control group (control); shH19 negative control (NC) group; shH19 group (shH19); puromycin (20 mg/l) group; shH19 + puromycin (20 mg/l) group (shH19 + puromycin); and Shensu IV (10 μg/ml) + puromycin (20 mg/l) group (Shensu IV + puromycin). The cells were treated with puromycin (20 mg/l) for 48 h. In the Shensu IV + puromycin group, the cells were first treated with 10 μg/ml Shensu IV for 24 h, and then with puromycin for 48 h. The expression levels of H19, DIRAS3, mTOR, and p-mTOR in podocytes and those of the autophagic markers LC3 and p62 were detected by fluorescence qPCR and Western blot.

### Western blotting

Total proteins were extracted from tissues or cells using a TriplePrep Kit (Cat. no. 28-9425-44; ReadyPrep; Cytiva) and collected after centrifugation at 10000×***g*** for 10 min. Protein concentration was determined using a BCA kit (Beyotime, Beijing, China). Thereafter, the proteins were separated by sodium dodecyl sulfate/polyacrylamide gel electrophoresis for 1–2 h and transferred to nitrocellulose membranes as previously described [20]. After blocking in 5% skimmed milk at room temperature for 2 h, the membranes were incubated with the following primary antibodies at 4°C overnight: mouse monoclonal anti-actin (TA-09, ZSbio, 1:2000), rabbit anti-LC3 (ab192890, Abcam, 1:2000), rabbit anti-p62 (ab109012, Abcam, 1:10000), rabbit anti-mTOR (ab32028, Abcam, 1:1000), and rabbit anti-p-mTOR (AF3308, Affinity, 1/1000).

### Statistical analysis

All data were analyzed using SPSS 19 (IBM, U.S.A.) and expressed as means ± standard deviation. One-way ANOVA followed by Bonferroni test was applied to determine statistical significance. A *P*-value <0.05 was considered statistically significant.

## Results

### The identification of the main components in Shensu IV

**Figure 1:**
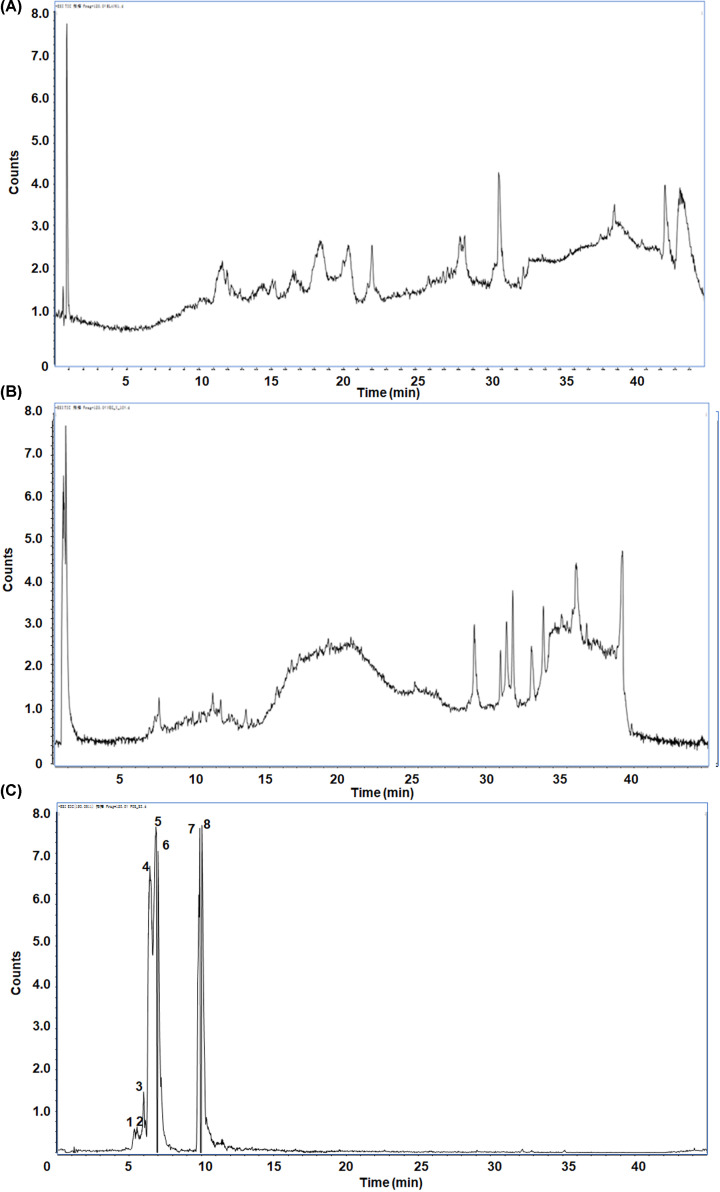
The main components in Shensu IV as detected by LC–MS (**A**) Negative ion mode total ion chromatogram (TIC). (**B**) Positive ion mode TIC. (**C**) Base peak ion chromatography and corresponding compounds. 1: l-Carnitine; 2: LPC 16:0; 3: Coumaroyl tyramine; 4: Tetramethylpyrazine; 5: LPC 18:1; 6: Choline; 7: (S,S)-Butane-2,3-diol; 8: Scopoletin.

Liquid chromatography–mass spectrometry (LC–MS) results indicated that the main active chemical substances in Shensu IV were l-Carnitine, P-lysoPC (LPC) 16:0, Coumaroyl tyramine, Tetramethylpyrazine, LPC 18:1, Choline, (S,S)-Butane-2,3-diol, and Scopoletin (Figure 1). The retention times, molecular weights, and MS data of the identified peaks are shown in Table 2.

**Table 2:** The retention time, molecular weight, and MS data of the identified peaks

No.	Compounds	RT (min)	MW	MS
1	l-Carnitine	0.77695	161.2	162.1129
2	LPC 16:0	26.34937	495.63	496.3426
3	Coumaroyl tyramine	13.11965	283.32	284.1285
4	Tetramethylpyrazine	4.503284	136.19	137.111
5	LPC 18:1	27.22265	521.3	522.3564
6	Choline	0.8071333	104.17	104.1091
7	(S,S)-Butane-2,3-diol	12.07172	90.12	73.06499
8	Scopoletin	6.421317	192.17	193.0511

### Shensu IV protected against pathological changes in rats with PAN-induced nephropathy

**Figure 2:**
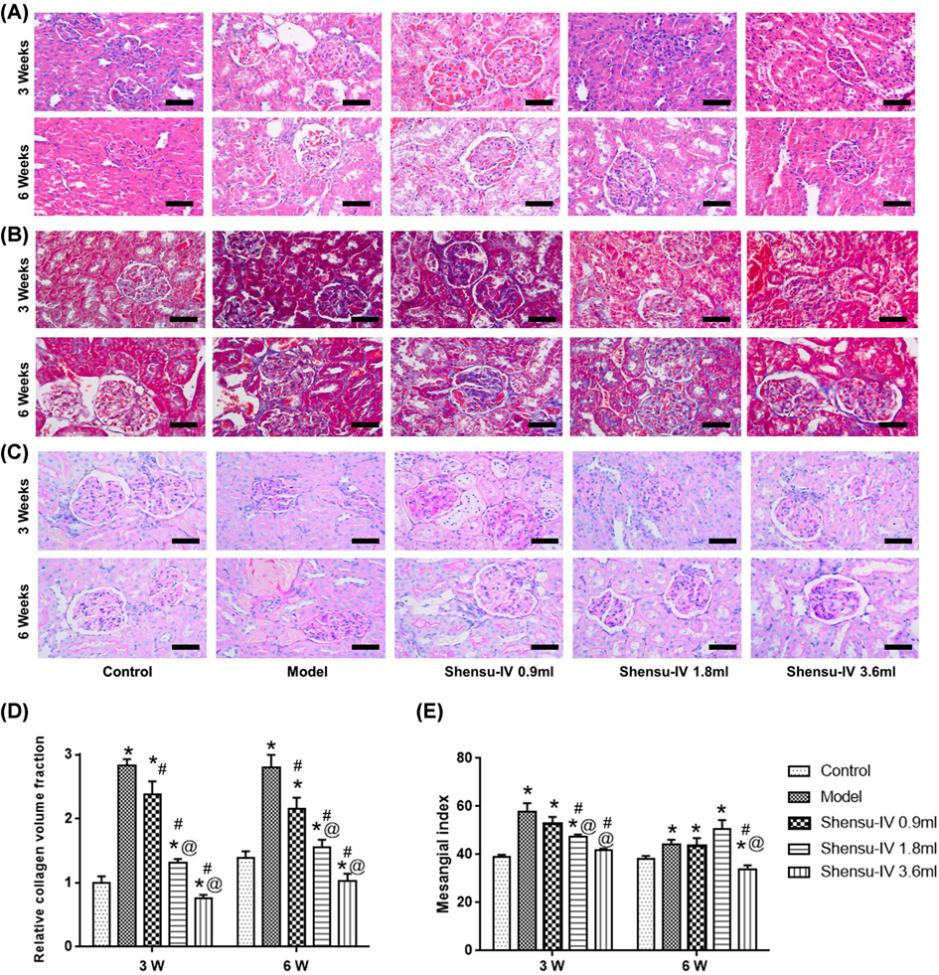
Pathological changes in kidney tissues (**A**) Hematoxylin and Eosin (H&E), (**B**) Masson’s, and (**C**) PAS staining were used to observe the pathological changes in renal tissues in each group. Magnification: ×200. (**D**) The relative collagen volume fraction. (**E**) The mesangial index. Compared with the control group, *P<0.05; compared with the model group, ^#^*P*<0.05; compared with the Shensu-IV 0.9 ml group, ^@^*P*<0.05.

The model of nephropathy was confirmed by testing the urine protein level of the rats. The pathological changes in kidney tissue were determined according to the relative collagen volume fraction and mesangial index (Figure 2). Compared with the model group, Shensu IV treatment exerted preventive effects, especially the medium and high doses.

**Figure 3:**
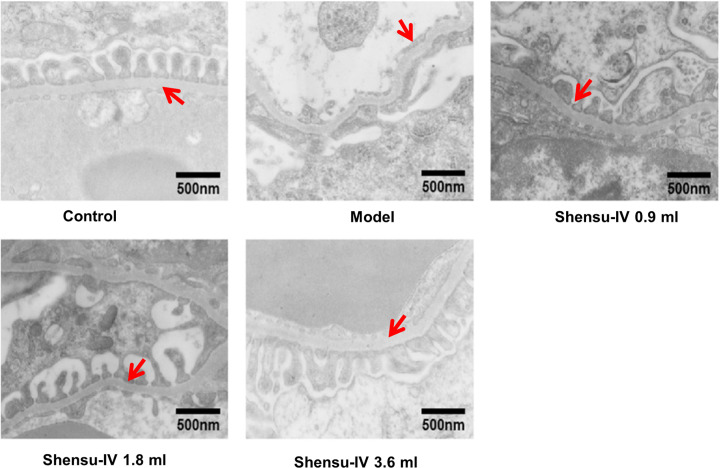
The effects of Shensu IV on glomerular podocytes of nephropathic rats In control group, the glomerular podocytes were arranged in a uniform and orderly manner, and the thickness of basement membrane was uniform and consistent. By contrast, the podocyte of glomerulus was widened, fused into plate, and the basement membrane was thickened locally in the model group. The ultrastructure was repaired by the treatment with high, middle, and low doses of Shensu IV. The arrows show the glomerular basement membrane.

In control group, the glomerular podocytes were arranged in a uniform and orderly manner, and the thickness of basement membrane was uniform and consistent. By contrast, the podocyte of glomerulus was widened, fused into plate, and the basement membrane was thickened locally in the model group. The ultrastructure was repaired by the treatment with high, middle, and low doses of Shensu IV (Figure 3).

### Shensu IV increased the level of autophagy in the podocytes of rats with PAN-induced nephropathy

**Figure 4:**
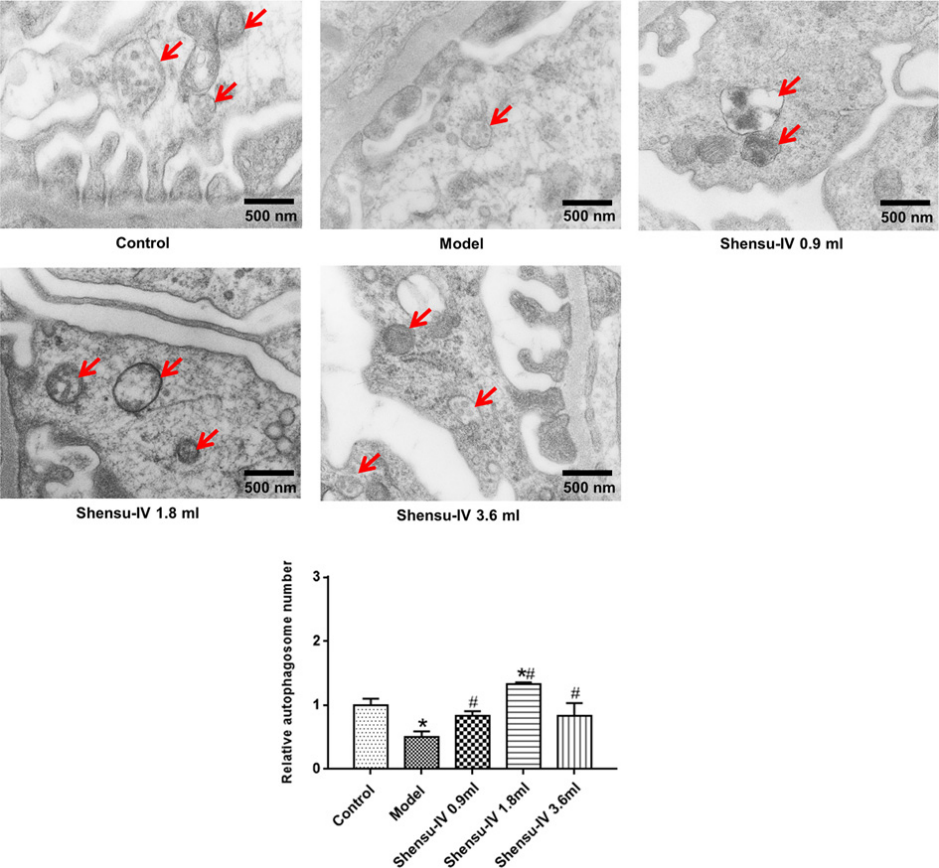
The effects of Shensu IV on the numbers of autophagosomes in podocytes of nephropathic rats The arrows show the autophagosome structure. Compared with the control group, *P<0.05; compared with the model group, ^#^*P*<0.05.

The level of autophagy in podocytes was determined using transmission electron microscopy. At the end of week 6, there were significantly fewer autophagosomes in the podocytes of the model group compared with that in the normal group. Additionally, the level of autophagy was greatly increased in podocytes of the treatment groups (high, middle, and low doses of Shensu IV) (Figure 4).

**Figure 5:**
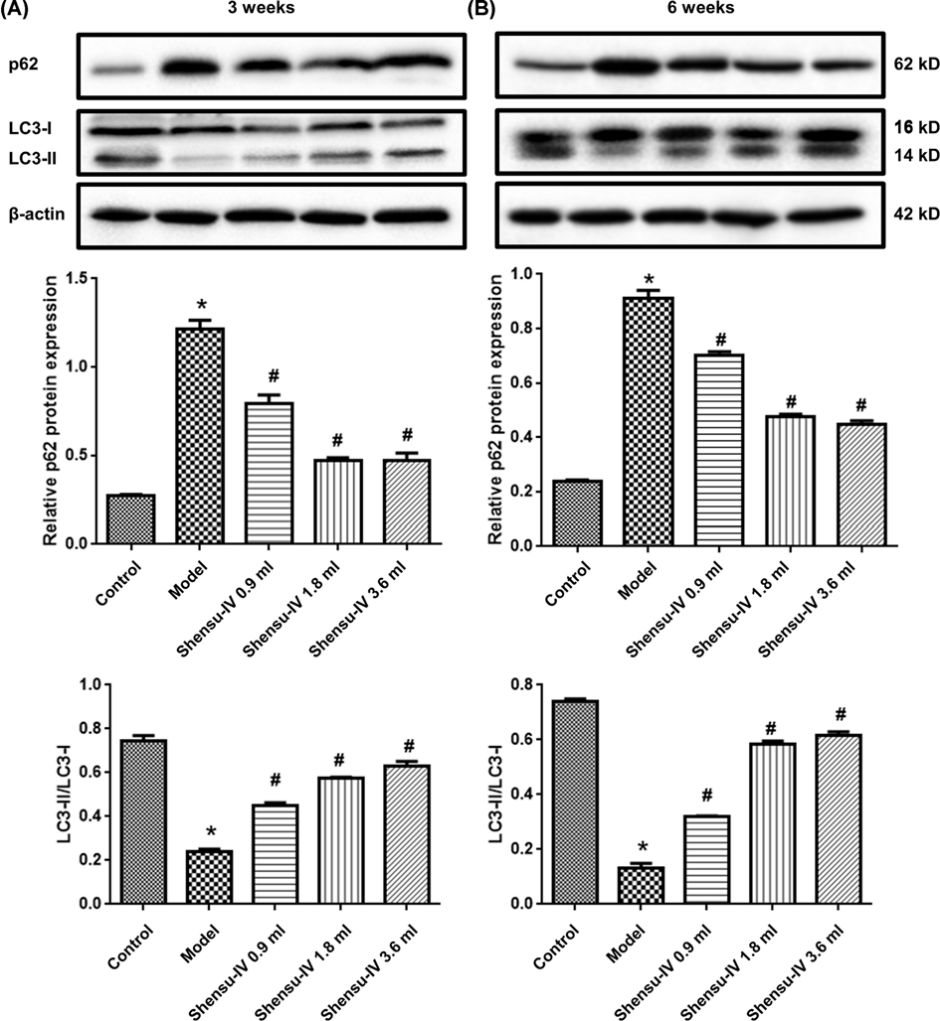
The LC3-II/LC3-I ratio and the expression of the p62 protein in kidney tissue (**A**) Three weeks after treatment and (**B**) six weeks after treatment. Original blots are shown in Supplementary Figure S1; compared with the control group, *P<0.05; compared with the model group, ^#^*P*<0.05.

**Figure 6:**
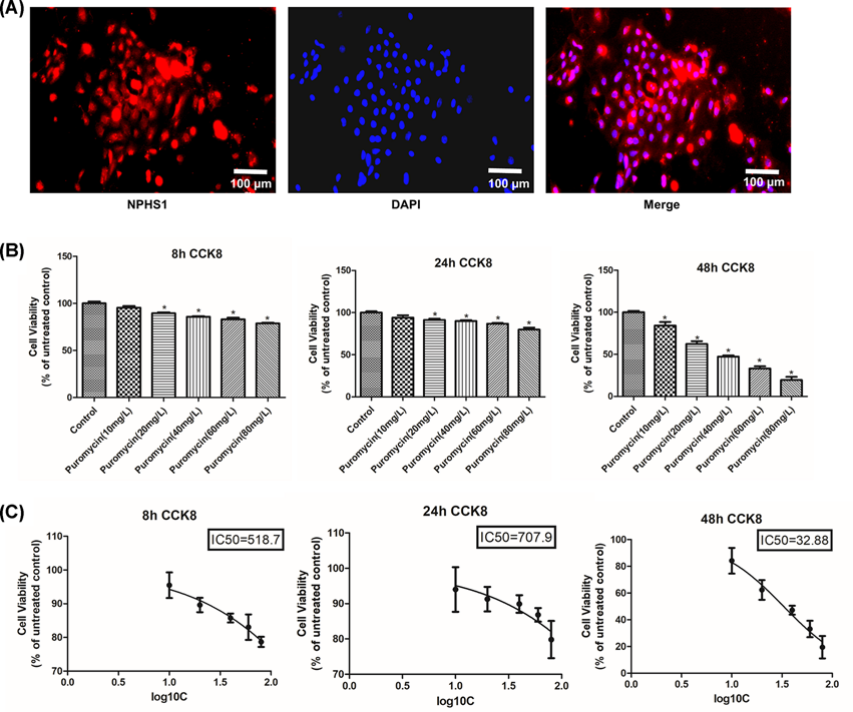
The identification of primary podocytes and the selection of the optimal puromycin concentration (**A**) The identification of primary podocytes by immunofluorescence. (**B**) The selection of puromycin concentration and treatment duration based on CCK8 assay. (**C**) The IC_50_ of puromycin at the 8, 24, and 48 h time points. Compared with the control group, *P<0.05.

The expression level of p62 was significantly higher in the model group than in the control group, whereas the LC3-II/LC3-I ratio was significantly lower (*P*<0.05), at both the 3- (Figure 5A) and 6-week time points (Figure 5B). Meanwhile, the expression level of p62 in the Shensu IV groups (high, middle, and low doses of Shensu IV) was significantly decreased at both time points (Figure 5A), whereas the LC3-II/LC3-I ratio was significantly increased (Figure 5B) (*P*<0.05 *vs.* the model group).

### Shensu IV exerted protective effects against puromycin-induced injury in podocytes

The podocyte identity of the isolated cells was confirmed through the positive expression of NPHS1 (Figure 6A). Cell proliferation and IC_50_ values were detected at 8, 12, and 24 h in podocytes treated with different concentrations of puromycin. Because we found that treatment with 20 mg/l puromycin for 48 h could inhibit podocyte proliferation, this concentration was selected to induce cell injury (Figure 6B,C).

**Figure 7:**
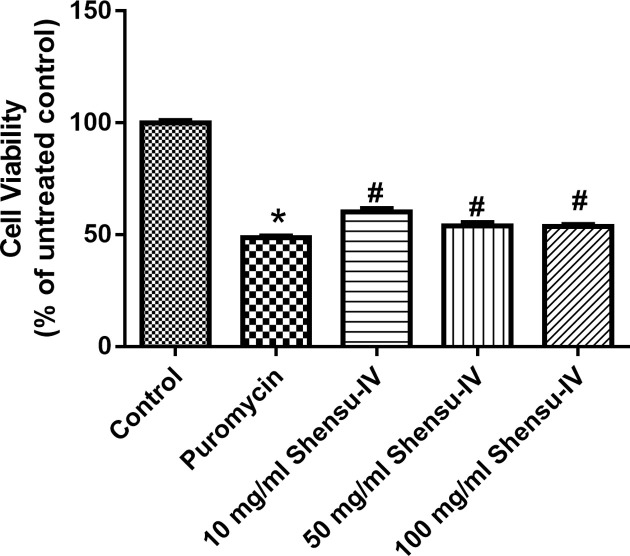
The effects of Shensu IV on cell viability of puromycin-treated podocytes Compared with the control group, *P<0.05; compared with the puromycin group, ^#^*P*<0.05.

Cell proliferation in the puromycin treatment group was significantly decreased compared with that of the control group (*P*<0.05); however, compared with the puromycin group, cell proliferation was significantly increased with Shensu IV treatment at the concentration of 10, 50, and 100 μg/ml (Figure 7). Consequently, 10 μg/ml was used in subsequent experiments.

### ShH19 and Shensu IV administration promoted autophagy in podocytes with puromycin-induced injury

**Figure 8:**
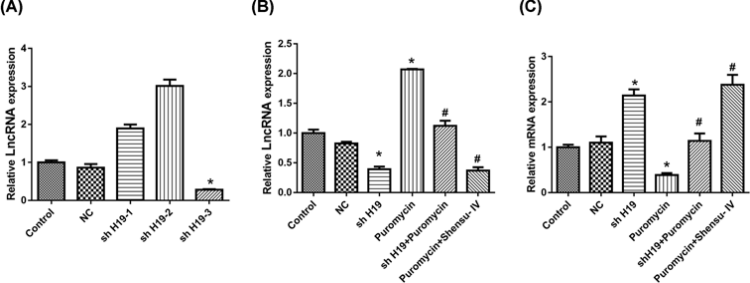
Treatment with shH19 or Shensu IV up-regulated the *DIRAS3* mRNA expression level (**A**) The effects of shH19 on H19 expression. (**B**) H19 expression after the respective treatments. (**C**) The *DIRAS3* mRNA expression level after the respective treatments. Compared with the negative control (NC) group, *P<0.05; compared with the puromycin group, ^#^*P*<0.05.

Compared with the control group, shH19-3 treatment resulted in a significant reduction in H19 expression (Figure 8A). As shown in Figure 8B, the expression of lncRNA H19 in the shH19-treatment group was significantly lower than that of the NC group. Puromycin promoted, whereas shH19 and Shensu IV reduced, the expression of lncRNA H19.

We also detected the expression of *DIRAS3* at the mRNA level. Compared with the NC group, shH19 treatment significantly up-regulated, whereas puromycin down-regulated the expression of *DIRAS3*. Both shH19 and Shensu IV increased the expression levels of *DIRAS3* in puromycin-treated cells (Figure 8C).

**Figure 9:**
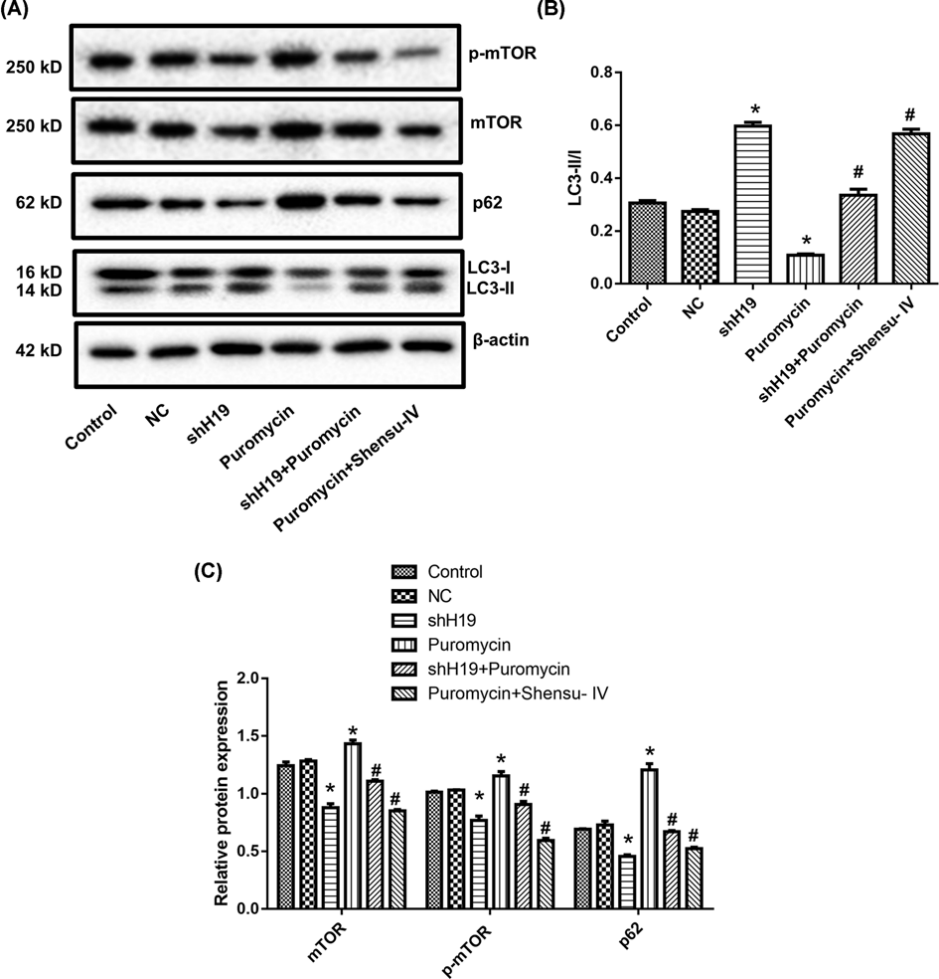
Treatment with shH19 or Shensu IV promoted autophagy in puromycin-induced injury in podocytes (**A**) Representative blots of mTOR, p62, and LC3. Original blots are shown in Supplementary Figure S1. (**B**) Quantification of the LC3-II/I ratio. (**C**) Quantification of the levels of mTOR, p-mTOR, and p62. Compared with the negative control (NC) group, *P<0.05; compared with the puromycin group, ^#^*P*<0.05.

Additionally, we measured LC3 and p62 expression by Western blotting (Figure 9). The ratio of LC3-II/I was increased in the shH19 group compared with that in the NC group. Puromycin treatment reduced the LC3-II/I ratio, whereas this ratio was increased with the application of shH19 or Shensu IV (Figure 9A,B). The expression of p62 showed the opposite trend. ShH19 reduced, while puromycin increased, p62 expression levels. Both shH19 and Shensu IV reduced the expression of p62 in puromycin-treated cells (Figure 9A,C).

ShH19 treatment reduced mTOR and p-mTOR expression, whereas puromycin elicited the opposite effect. Both shH19 and Shensu IV reduced p62 expression in puromycin-treated cells (Figure 9A,C).

## Discussion

Podocytes are specialized epithelial cells with limited regenerative ability and are one of the components of the glomerular filtration barrier [2]. Podocyte injury contributes to the development of several nephropathies, and is closely associated with proteinuria and glomerulosclerosis [21]. In this study, a PAN solution was injected into the jugular vein of rats to establish a rat model of nephropathy. A urine protein level that ranged between 3 and 5 g/l indicated that the model had been successfully established. Shensu IV can reduce proteinuria and improve serum albumin levels in patients with chronic glomerular disease. Additionally, Shensu IV can reportedly alleviate mesangial cell proliferation, mesangial matrix accumulation, tubular atrophy, and renal interstitial fibrosis, and can also protect against glomerular podocyte injury in rats with PAN-induced nephropathy [15]. In this study, Hematoxylin and Eosin (H&E), Masson’s, and PAS staining were used to detect pathological changes in renal tissues. The results showed that rats in the model group displayed typical nephropathy-related pathological changes, which were improved by Shensu IV administration.

Autophagy plays an important role in maintaining podocyte stability [22]. Mice with podocyte-specific deletion of the autophagy-related gene *Atg5* exhibit reduced autophagic activity in podocytes, concomitant with the accumulation of oxidized protein, endoplasmic reticulum stress, and proteinuria, which eventually leads to podocyte injury and glomerulosclerosis [23,24]. In cultured podocytes, sc5b-9-mediated injury enhances podocyte autophagic activity, thereby playing a protective role, suggesting that enhancing autophagy may represent a strategy for the treatment of immune-mediated podocytosis [25]. The level of glomerular autophagy is reduced in rats with diabetic nephropathy compared with controls. Insulin treatment can increase the level of autophagy and reduce glomerular-associated pathological changes and podocyte injury, suggesting that the increased level of autophagy may help delay the progression of diabetic nephropathy [26]. Shensu IV has previously been reported to promote autophagy [15]. In the present study, autophagic activity was detected in podocytes of control rats. In the early stage of PAN-induced podocyte injury, the number of autophagosomes in podocytes decreased significantly; however, the administration of Shensu IV could reverse this effect, indicating that Shensu IV exerted a regulatory effect on podocyte autophagy in rats with PAN-induced nephropathy.

Numerous proteins are involved in the autophagic pathway, including LC3 and p62. LC3 is an important marker of autophagy; when autophagy occurs, it is converted from a type I (LC3-I) into a type II [27]. P62, which serves as an autophagy substrate, is negatively regulated by autophagy and is also widely used to evaluate autophagic levels [28]. In this study, Western blotting was used to detect the expression of LC3 and p62 in glomerular podocytes. We found that the expression level of LC3-II/LC3-I ratio in podocytes of the treatment group was significantly higher than that of podocytes of the model group, whereas that of p62 was significantly lower. Autophagy was inhibited in the early stage of PAN-induced podocyte injury; however, the expression level of LC3-II in the model group was still significantly lower than that of the control group at the end of week 6, indicating that PAN can inhibit autophagy in glomerular podocytes for extended periods, which may lead to sustained podocyte damage.

To further elucidate the molecular mechanism underlying how Shensu IV regulates podocyte autophagy in rats with PAN-induced nephropathy, we isolated podocytes from SD rats. Podocyte identity was confirmed through the positive expression of NPHS1. PAN was used to establish a model of podocyte injury *in vitro*. In addition, a CCK8 assay was used to select the appropriate exposure duration and concentration of puromycin, which were found to be 48 h and 20 mg/l, respectively. Also using CCK8 assay, we identified 10 μg/ml as the optimal concentration for Shensu IV treatment.

H19 is highly expressed in endodermal, mesodermal, and differentiated tissues during embryonic development [29]. It is not expressed in other tissues, except for the heart and skeletal muscle in the postnatal period. H19 can only be reactivated under conditions of tissue damage, tissue repair, stress, and tumorigenesis [30]. LncRNA H19 can act either as an oncogene or a tumor suppressor gene, and is reported to play an important role in the occurrence, development, invasion, and metastasis of various tumors, and can also serve as a biomarker of cancer prognosis [31,32]. LncRNA H19 can inhibit autophagy in a cell model of cerebral ischemia–reperfusion (OGD/R) [33]. Moreover, the H19/DIRAS3 pathway can modulate high glucose-induced autophagic activity in cardiomyocytes. Increased expression of H19 can down-regulate the expression of *DIRAS3*, thereby promoting mTOR phosphorylation, which inhibits the autophagic activity of cardiac muscle cells [34]. In our study, shH19 was used to knock down H19 expression, and showed an efficiency greater than 70% based on qPCR analysis. Evidence suggests that DIRAS3 is involved in the regulation of autophagy, increasing the formation of autophagic vesicles by promoting ATG4 expression. DIRAS3 can also regulate autophagic activity through the PI3K/AKT/mTOR and AMPK/TSC1/TSC2 signaling pathways [35]. Additionally, the expression of DIRAS3 is up-regulated under conditions of malnutrition, which leads to the induction of autophagy [36]. In this study, we measured the expression levels of H19 and *DIRAS3* by quantitative real-time reverse transcription-PCR (RT-qPCR). Our data indicated that puromycin treatment significantly increased the expression of H19 in podocytes, while decreasing that of *DIRAS3*. However, these expression patterns were altered following treatment with either shH19 or Shensu IV. These data suggested that Shensu IV can promote glomerular podocyte autophagy by interfering with the expression of lncRNAH19/DIRAS3, and thereby exerting protective effects on podocytes.

The mTOR signaling pathway is an important regulator of the autophagic balance in podocytes [37]. The reduction in podocyte autophagic activity in rats with PAN-induced nephropathy was closely related to the activation of the mTOR signaling pathway. Shensu IV can effectively inhibit mTOR pathway activation, thereby regulating the balance of podocyte autophagy, and, consequently, preventing podocyte damage [15]. The lncRNA H19/DIRAS3 pathway can regulate autophagy through the mTOR signaling pathway [34]. Here, we measured the expression levels of mTOR and p-mTOR in podocytes. We found that mTOR and p-mTOR expression was significantly increased in podocytes treated with puromycin; however, the opposite effect was observed with shH19 and Shensu IV treatment. These results suggested that Shensu IV can regulate mTOR signaling through its effects on lncRNAH19/DIRAS3, thereby promoting glomerular podocyte autophagy and inhibiting podocyte damage.

In conclusion, our data showed that the lncRNAH19/DIRAS3 pathway is involved in podocyte autophagic activity and that Shensu IV can regulate the autophagic balance in podocytes. We also found that Shensu IV exerts protective effects on glomerular podocytes by promoting autophagy in an lncRNAH19/DIRAS3/mTOR-dependent manner.

## Supplementary Material

Supplementary Figure S1

## Data Availability

The datasets used during the present study are available from the corresponding author upon reasonable request.

## Abbreviations

CCK8: Cell Counting Kit-8
DAPI: 4′,6-diamidino-2-phenylindole
DIRAS3: distinct subgroup of the RAS family member 3
LC–MS: liquid chromatography–mass spectrometry
LC3: Microtubule-associated protein 1A/1B-light chain 3
lncRNA: long non-coding RNA
LPC: P-lysoPC
PAN: puromycin aminonucleoside
PAS: Periodic Acid–Schiff
PFA: paraformaldehyde
qPCR: Quantitative Real-time PCR
SD: Sprague–Dawley
