# Supplementary figures and images for: Shensu IV prevents glomerular podocyte injury in nephrotic rats via promoting lncRNA H19/DIRAS3-mediated autophagy
Source: Biosci Rep. 2021 May 4;41(5):BSR20203362. doi: 10.1042/BSR20203362 (PMC8112846; doi:10.1042/BSR20203362)

sFig.1 Original blots for Figure.4 and Figure.9A.

Figure.5

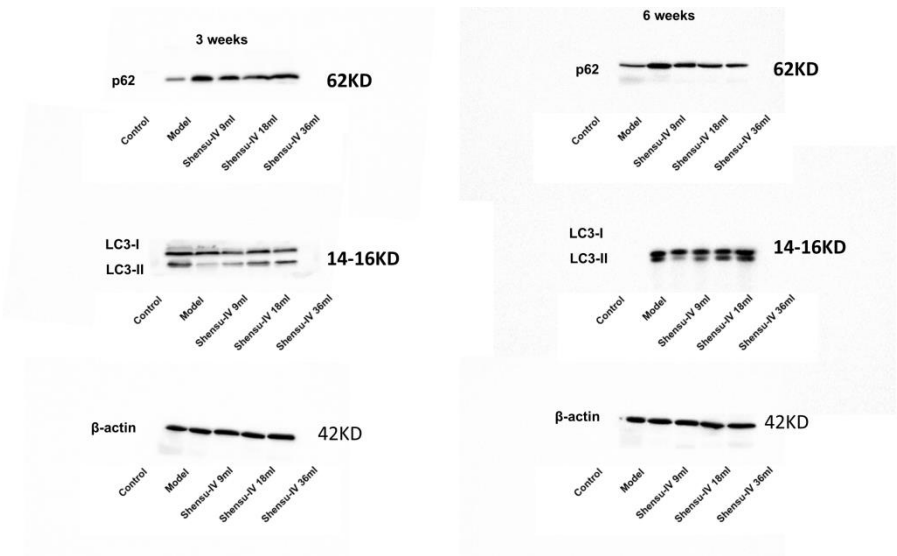

Figure.9A

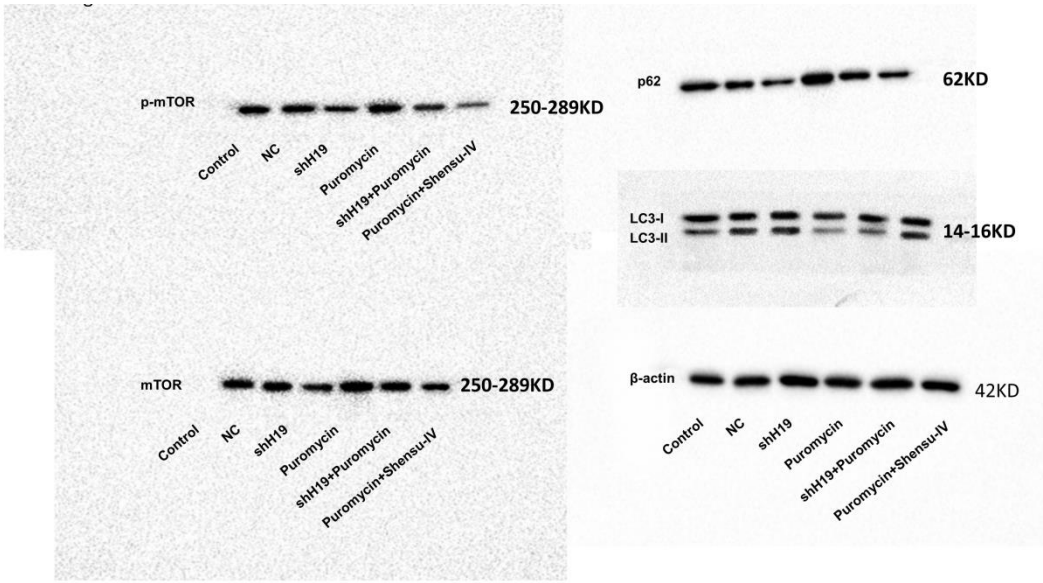

Supplement: Supplementary Figure S1 [file BSR-2020-3362_supp.pdf]
